# Changing vulnerability in Asia: contagion and spillovers

**DOI:** 10.1007/s00181-022-02322-5

**Published:** 2022-11-04

**Authors:** Moses Kangogo, Mardi Dungey, Vladimir Volkov

**Affiliations:** Tasmanian School of Business and Economics, University of Tasmania, Private Bag 84, Hobart, TAS 7001 Australia

**Keywords:** Financial stability, Financial networks, Asian markets, Financial crises, Spillover and contagion, G15, C21, N25, G01

## Abstract

An increasing involvement of the Asian market in the global context plays a fundamental role in spreading shocks across the financial system. This paper examines the extent of vulnerability across Asian equity markets and the United States (US) equity market by distinguishing between spillovers and contagion. Spillovers are detected using a generalised historical decomposition method, while contagion is identified using a portfolio mimicking factor framework using moment conditions. The transmission of spillovers is assessed to capture the direction, strength and signs of the spillovers. The findings show evidence of changing vulnerability in Asia and the US. This is as a result of increased spillovers during crisis events and the presence of contagion. Stronger connections during crisis periods are evident as well as a general deepening of the global network. These connections may result in reduced opportunities for emerging markets. The findings suggest that caution is needed when developing regulations or methods to create a stable financial system.

## Introduction

The unexpected occurrence of crisis events such as the Asian financial crisis of 1997–1998 and the global financial crisis of 2007–2009 pose great threat to the stability of the economy. A common threat throughout these events is the transmission of shocks from one market to another. As global financial markets are increasingly globalised and integrated, the shocks from one market is very likely to be transmitted to other markets through various mechanism that include trade, credit and balance sheet channels. The magnitude of these transmissions may increase during crisis periods causing financial instability.

The main objective of this paper is to investigate the impact of shock transmissions in financial markets with specific emphasis on the Asian markets. This is achieved by distinguishing the roles of spillovers and contagion in destabilising the economy. The distinctions between spillovers , contagion, decoupling and interdependence are important for designing policies for financial stability. Allen and Wood (2006) discuss how to determine the appropriate speed of adjustment in markets. An asymmetric policy response may be needed to capture only the shocks that are going to have negative effects on the recipient economy. In different circumstances, spillover, contagion, or decoupling could either be undesirable or have useful outcomes. The problem is similar to that of research and development spillovers where there are offsetting effects from having rivals in product markets and technology spillovers (Lucking et al. 2018). Lucking et al. (2018) conclude that the positive aspects of research and development spillovers overwhelm the negative in welfare analysis.

Motivated by the increasing role of the Asian markets, this paper investigates the changing vulnerability over time to detect evidence for contagion and the time evolution of spillovers from the global market affecting the Asian markets and compare this evidence with regionally sourced influences. We differ from the existing literature that detects either contagion and spillover independently, by focusing on detecting contagion and spillover simultaneously and considering the influence of Chinese and US markets. Using both approaches to detect vulnerability in the global financial network provides more insights on the transmission channels of crisis events. This guides policy makers and regulators on developing appropriate policies which promotes financial stability. The US markets have been used as a proxy for global conditions in existing studies, such as Chiang et al. (2007) and Kim et al. (2015). Dungey et al. (2015) compared the influences of China and the US. Kim et al. (2015) argued vigorously against including China as a source of spillovers and contagion in financial market integration studies because of a perceived lack of market freedom in determining observed outcomes. Arslanalp et al. (2016) examined the growing role of spillovers from China to other Asian financial markets. Yilmaz (2010) tested whether the inclusion of India and China is important for calculating a spillover index for the region; they found that the impact was evident only after 2002. We estimated shocks transmissions by implementing the recently developed spillover and contagion methods to detect and measure spillovers and contagion. The spillover method builds on the index developed by Diebold and Yilmaz (2009, 2014), which provides a summary measure of financial spillovers in a network of markets based on a forecast error variance decomposition of a vector autoregression (VAR) of returns data. The Diebold–Yilmaz (DY) connectedness index has attracted a great deal of attention in the literature as a means of determining building pressure in spillovers between markets. The index was applied in Diebold and Yilmaz (2009, 2012, 2014, 2015), Demirer et al. (2018) and Yilmaz (2010) among others. Dungey et al. (2018b) showed that by rearranging information in the same VAR structure, it is possible to obtain information on the source of the spillovers affecting each market and the extent to which spillovers from one market affect others, and to distinguish these effects with signs. This makes it possible to distinguish whether the spillover is associated with either positive or negative shocks.

Identifying positive and negative spillover effects is important because it allows assessment of whether transmissions via spillovers amplify or dampen shocks originating in one market and affecting others. In general, links that amplify the transmission of bad shocks to other markets are undesirable during crises periods. We argue that these are the shocks policy makers should be most concerned about. To do this, it is important to be able to distinguish between amplifying shocks and dampening shocks. This means that when a shock from one market is dampened in its transmission, it contributes to the usually desirable outcome of reducing volatility in the recipient market. Dampening shocks lead to undesirable outcomes if paths that provide counter-balancing measures are inadvertently shut down. These counter-balancing measures aim to stop harmful transmission paths of shocks in the financial system. For this reason, we introduced a time-varying measure of both the size and direction of contributions of spillovers to the transmission of shocks between markets.

This paper uses the Dungey and Renault (2018) contagion tests and compares the outcomes with the traditional Forbes and Rigobon (2002) uncorrected and corrected tests. We also identify whether the tests are consistent with contagion, interdependence or decoupling, moving beyond the one-sided contagion test common in the correlation test literature. We consider three aspects of recent developments in the literature on modelling transmissions between markets during periods when turmoil appears and disappears in other markets. We contribute to the literature by investigating how vulnerability changes over time and highlighting the role of Asian markets. We focus on the impact of shock transmission on Asian markets and specifically incorporate the following:

i. modelling the time-varying contribution of spillovers for Asian markets during and after the GFC,
ii. distinguishing between amplifying and dampening transmissions in spillover linkages, and between contagion, interdependence and decoupling.

We investigate the changing nature of shock transmission in the financial system by subdividing our sample into four phases namely the pre-GFC representing the lead-up to the global financial crisis, the GFC representing the global financial period, the EDC representing the European debt crisis and the most recent period. Our results show that financial markets are exposed to large shocks during crisis periods. The US continues to transmit large shocks to the Asian markets. Results shows the emerging role of China in transmitting shocks to other financial markets. Our results also show evidence of strong contagion from the US to the Asian markets during the global financial crisis. Caution need to be taken in monitoring the financial markets in order to ensure stability in the financial system. Overall, we find strong evidence of the changing vulnerability in the global market during the different subperiods. Large shocks are transmitted and absorbed from one market to another during crisis periods. These results are important in providing guidance in designing appropriate policies to monitor these financial markets.

The remainder of this paper is organised as follows. Section 2 provides a brief review of the related literature of spillovers and contagion. Section 3 describes our data sample. Section 4 discusses the methodology on detecting contagion and spillovers. Section 5 presents the empirical analysis and discusses the results. Section 6 discusses implications of the results, while Sect. 7 concludes.

## Literature review

Our paper is closely related to the literature on detecting the changing nature of transmissions of shocks. A popular approach is to use correlation-based tests to detect unexpected changes in transmission from Asian markets to international markets, where Asian markets are used as the source of contagious shocks. This is particularly true during the Asian financial crisis. The literature on this includes Forbes and Rigobon (2002), who used Hong Kong and China as the source of shocks to other markets in a bivariate correlation framework; Sander and Kleimeier (2003) searched for contagion within Asia and from Asia to other emerging markets using Granger causality tests. Baur and Schulze (2005) considered quantile regressions in a co-exceedance framework to detect shocks from Thailand and Hong Kong to other Asian and international markets. Finally, Baur and Fry (2009) used both cross-section and time-series identification to estimate the spread of contagion within Asian markets. Much of the literature on measuring contagion from the Asian financial crisis is reviewed in Dungey et al. (2005). Since then, new methods have emerged and have been tested on the dataset for the Asian financial crisis. These methods include the generalised autoregressive conditional heteroskedasticity (GARCH) process (Dungey et al. 2015), dynamic conditional correlation (DCC) (Chiang et al. 2007), smooth transition, indices and other time-varying models (Kim et al. 2015) and copulas (Busetti and Harvey 2010).

A smaller body of literature concentrates on how the Asian markets were affected by shocks originating elsewhere. Examples include Hwang et al. (2013) and Kim et al. (2015) who considered the impact of the US financial crisis on emerging markets. Kim et al. (2015) also drew attention to the importance of examining this issue for interventions to protect Asian economies from crises emanating elsewhere. ADB (2017) investigated whether crises from other economies affect Asian economies. Beirne et al. (2010) considered local, regional and global effects for 41 emerging markets and concluded that significant spillovers from global effects cannot be ignored in Asian markets. Mobarek et al. (2016) used all possible pairings between 10 emerging and 10 developed markets, including seven Asian markets, in a DCC mixed-data sampling framework. They conclude that there are many different and time-varying relationships between them that will affect the efficacy of policy making. These multivariate approaches are typically based on equity market data and either consider particular subgroups of countries or bundle Asian markets together.

The increasing importance of Asian financial markets in the global context especially China has led to a growth in the literature focusing on spillovers between financial markets in Asia and other markets, both regional and international. Spillovers are the normal flow of information and adjustment of portfolios between markets, although this does not imply that spillovers are static. Yilmaz (2010) provided a time-varying spillover index for East Asian markets. Spillovers do not capture the abrupt changes associated with stress caused by contagion. Rather, they evolve relatively slowly with increasing financial integration, trade relationships, and the normal course of business and expansion. The literature comparing these types of channels includes Van Rijckeghem and Weder (2001) and Dungey et al. (2018). Given the growth in the size and relative importance of Asian markets, we believe that the relationships between Asian and global financial markets have changed since the start of the twenty-first century in response to changing cross-regional relationships and periods of financial stress experienced during crises.

### Brief overview of contagion

Contagion effects were considered to have negative impacts in the early literatures, such as Forbes and Rigobon (2002). The contagion effect introduced by Forbes and Rigobon (2002), as a one-sided test in asset returns among financial markets, is associated with statistically significant increases in correlation beyond what would be expected during normal conditions, even after controlling for increased market volatility. This increased volatility is regarded as undesirable because it can lead to flight to quality, leverage effects and a flight to home or a flight to familiarity. A flight to home and a flight to familiarity can be attributed to increased risk and uncertainty in both markets experiencing crisis and those associated with them (Giannetti and Laeven 2015). Arguably, the most important empirical debate in the literature has been to distinguish periods of contagion from interdependence due to changes in volatility in periods of stress in the financial system.

An appealing way of testing for contagion is via changes in correlation between assets or markets. A correlation coefficient is a simple transformation of the links between two markets, scaled by their relative volatility (i.e. in the regression of $y_{t} = \beta x_{t} + \varepsilon _{t}$, where *y* and *x* are stochastic variables representing different stock market returns, and $\beta$ is the ordinary least square (OLS) estimate and $\varepsilon _{t}$ residuals. The correlation coefficient is given by $\rho = \beta \sigma ^{x} \sigma ^{-y}$ where $\sigma ^{x}$ is the variance of *x* and $\sigma ^{y}$ the variance of *y*). A simple test of change in transmission between two sample periods is to test whether $\rho _{1} = \rho _{2}$, which is essentially a proxy for the underlying test of $\beta _{1} = \beta _{2}$ (where $\rho _{1}$ and $\rho _{2}$ are the correlation coefficients in the two periods, and $\beta _{1}$ and $\beta _{2}$ are the OLS estimates in the two periods.). Forbes and Rigobon (2002) asserted that there is a mechanical relationship between increased volatility and increases correlation coefficient between periods. They suggest a scaled version of the correlation coefficient to correct the test. Empirically, this vastly reduces the incidence of contagion identified between the uncorrected and corrected correlation tests. Hence, the Forbes and Rigobon (2002) correction has been shown to be overzealous and results in the under-detection of contagion. This is partly due to the need to accommodate the bounded nature of correlation coefficients in applying t-tests to the difference between them via a Fisher correction. Dungey and Zhumabekova (2001) examined the properties, and Dungey et al. (2005) examined a correction. However, even this relies on unconditional variance estimates for distinct periods.

Two developments have provided some improvement for contagion detection. The first is the implementation of two-sided tests, in which contagion is associated with statistically significant increases in transmission links (correlation) between assets. Here, when there are no statistically significant changes, it is labelled interdependence; evidence of a statistically significant reduction in the transmission of shocks between assets (correlation) is labelled decoupling. Decoupling stems from the literature, including Caporin et al. (2018), who showed that Portugal’s and Greece’s debt markets during the European debt crisis were less associated with movements in source markets than they were during normal times. Evidence of these effects is becoming more pronounced, particularly as studies of financial markets under stress consider a greater variety of potential links with the use of multivariate models and increased processing capacity for higher-order models.

The second development is the use of conditional variance to identify contagion effects, and thereby control for changes in the relative volatility of the assets under consideration. Contagion tests in the correlation form implicitly rely on the assumption that the relative contribution of idiosyncratic and market shocks remains the same for each asset during periods of stress and calm. Using a decomposition that takes advantage of the conditional variance of the assets, Dungey and Renault (2018) showed how the underlying test of changes in transmission (contagion) between markets can accommodate the potential for change in the idiosyncratic volatility for individual assets. This changes the results in a priori unpredictable direction compared with the unconditional test results.

## Dataset and stylised facts

The dataset includes 12 Asian daily equity market indices (in local currencies) and the equity market index of Australia and the US for January 2003–December 2017 (see Table 1). These are daily (closing) equity market indices. We focused on Asian markets because of their growing importance in global financial markets.^1^ Specifically, we investigated the changing vulnerability among Asian markets and the rest of the world.

**Table 1:** Markets in the sample

Market	Abbreviation	Stock index	Market	Abbreviation	Stock index
Australia	AU	S &P/ASX 200	Philippines	PH	Philippine SE I (PSEi)
China	CN	Shanghai SE Composite	Singapore	SG	Straits Times Index
India	IN	S &P BSE National 200	South Korea	KR	Korea SE KOSPI 200
Indonesia	ID	IDX Composite	Sri Lanka	LK	Colombo SE All Share
Japan	JP	NIKKEI 225 Stock Average	Thailand	TH	Bangkok S.E.T
Hong Kong	HK	Hang Seng	Taiwan	TW	Taiwan SE Weighted TAIEX
Malaysia	MY	DJGL Malaysia	US	US	S &P 500 Composite

**Fig. 1:**
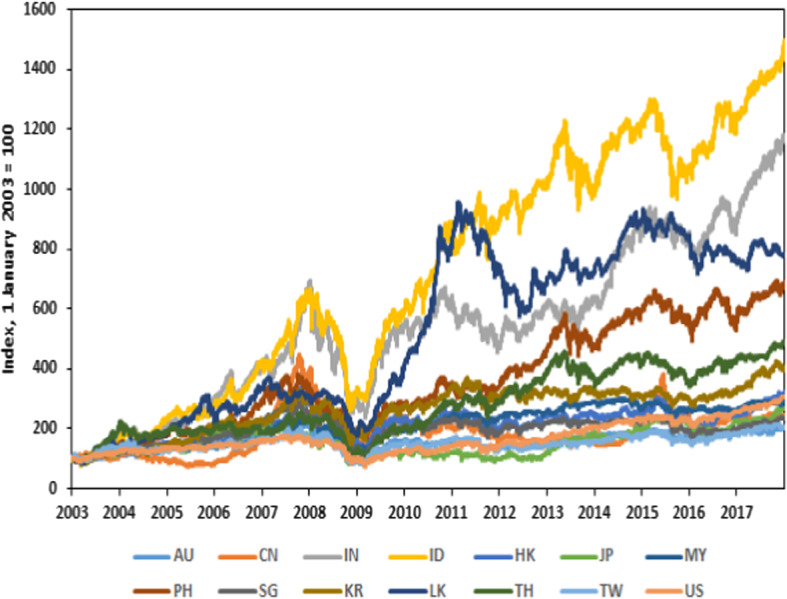
Equity market indices. The sample period is January 2003–December 2017. The source of the data is Thomson Reuters Datastream

Figure 1 plots the equity market indices for each market scaled such that the first observation is 100 in each series. Unit root tests revealed the usual characteristics of stationary returns in each series. The analysis was conducted using demeaned returns because the mean is usually extremely close to 0 and since we focus on decompositions, this assumption is innocuous. We used the data with their recorded closing time date.

Table 2 presents the descriptive statistic of the daily returns for each market. The mean returns are positive for all economies with standard deviation ranging from 0.0071 to 0.0156. The kurtosis results suggest that the daily return would be ‘peaked’ and have ‘fat-tailed’ distribution. Unit root tests revealed the usual characteristics of stationary returns in each series.

**Table 2:** Descriptive statistics of daily return for each market

Country	Mean	Min	Max	SD	Kurtosis	Skewness	ADF test	No. obs
AU	0.0002	− 0.0834	0.0579	0.0101	8.4716	− 0.3371	− 64.3072**	3913
CN	0.0004	− 0.0884	0.0946	0.0156	7.7914	− 0.3611	− 61.8116**	3913
IN	0.0007	− 0.1187	0.1631	0.0140	13.8639	− 0.1880	− 57.6653**	3913
ID	0.0008	− 0.1038	0.0792	0.0128	10.4305	− 0.4710	− 56.3705**	3913
HK	0.0004	− 0.1270	0.1435	0.0141	14.2746	0.2687	− 64.0384**	3913
JP	0.0004	− 0.1141	0.1415	0.0145	11.3534	− 0.3039	− 64.3730**	3913
MY	0.0003	− 0.0942	0.0460	0.0071	17.5838	− 1.0183	− 54.7780**	3913
PH	0.0006	− 0.1278	0.0981	0.0131	9.5235	− 0.3061	− 56.5428**	3913
KR	0.0004	− 0.1033	0.1223	0.0130	9.8787	− 0.2265	− 61.8854**	3913
SG	0.0003	− 0.0936	0.0717	0.0111	9.7341	− 0.0754	− 62.0741**	3913
LK	0.0006	− 0.1297	0.1231	0.0105	25.6197	− 0.4521	− 53.6479**	3913
TH	0.0005	− 0.1484	0.1116	0.0122	15.7426	− 0.6088	− 62.0660**	3913
TW	0.0003	− 0.0670	0.0672	0.0125	6.5743	− 0.1801	− 60.7618**	3913
US	0.0003	− 0.0903	0.1158	0.0113	15.6823	− 0.0942	− 69.4808**	3913

The sample period is January 2003–December 2017. The augmented Dickey–Fuller (ADF) statistic tests for unit root

**Indicate statistical significance at a 5$\%$ level

The US data are non-overlapping with Asian market timing so that events in the US on a given date cannot provoke a reaction in an Asian market until the following day.

In the contagion analysis, we lagged US returns by one day (with sensitivity tests against contemporaneous returns).

## Detecting contagion and spillovers

We begin by examining the time-varying nature of the contributions of shocks from the different sources over the sample period using an unconditional analysis to identify spillovers. We then consider the conditional relationships between markets during different periods in the sample. We use this to identify the extent of change in the propagation of shocks from source markets to target markets in different periods. These two approaches have several advantages over those in the literature. First, the effects of one market on another are signed. Through this approach, we are not only able to detect whether there is a significant transmission path of unusual shocks between markets, but we can also determine whether that transmission amplifies or dampens the effects on the recipient market. This aspect is not addressed in most studies that analyse shock transmissions (e.g. Diebold and Yilmaz 2009, 2014; Billio et al. 2012) and contagion (Forbes and Rigobon 2002). The extant literature primarily seeks evidence of significant links (and perhaps their direction) rather than the sign of those links. For policy and investment management purposes, however, the significance, direction and sign of the links are all relevant. Policy makers and investors want to know whether an event in a source market is likely to affect another market (via significance and direction) and whether that is likely to amplify or dampen volatility or returns (via sign) in the target market. We now introduce the two methodologies that enable us to assess these effects: generalised historical decomposition (GHD) methodology and contagion methodology.^2^

### Spillovers using the generalised historical decomposition methodology

Consider *n*-dimensional random vectors of returns from different markets, $r_{t}$, which we consider are related to each other in the normal course of internationally linked financial markets. We applied standard VAR to the random vector which is expressed as:

1 $$\begin{aligned} r_{t} = \Phi _{0} + \sum _{j=1}^{p}\Phi _{j} r_{t-j} + \varepsilon _{t} \end{aligned}$$

where *p* is the number of lags,^3^$\Phi _{j}$ and $\Phi _{0}$ are parameters of the model and $\varepsilon _{t}$ represents reduced form errors. There are many potential problems with modelling daily returns in this manner, including the issue of GARCH and non-normality. For example, Dungey et al. (2015) for discussion on the inclusion of GARCH into VAR representations. The problem is one of tractability—accounting for multivariate GARCH—greatly reduces the tractability of the model and increases its numerical complexity for estimation. In keeping with the approach of Diebold and Yilmaz (2009, 2014), we push these issues aside for the purposes of computing the spillover and directional spillover indices proposed here.^4^

Spillovers are measured by the combined effects of shocks originating in one market and spreading to other markets. That is, they represent how effects flow from one market to another. In the DY approach, the spillover measure is achieved using the forecast error variance decomposition matrix from the VAR at a specified forecast horizon. They obtain a time-varying measure by using VARs estimated from rolling windows of data across the sample. Thus, the DY spillover index involves two ex-ante modelling choices: the forecast horizon and the size of the rolling window.

The GHD takes the estimated VAR in a slightly different organisational direction. Rather than focusing on the forecast error variance decomposition, it uses the moving average representation of the reduced form VAR(p) to recognise that at any point in time (*t*). The reduced form VAR(p) can be rewritten in terms of disturbances conditional on the initial values as:

2 $$\begin{aligned} r_{t} = K_{t} + \theta (p)\varepsilon _{t} = K_{t} + \sum _{j=0}^{\infty }\theta _{j}\varepsilon _{t-j} \end{aligned}$$

where $\theta (p)$ is a matrix of polynomials in the lag operator *p* and $K_{t}$ is a function of those initial values. Any individual element $r_{i,t}$ can be represented by contributions of all variables as:

3 $$\begin{aligned} r_{i,t} = K_{t} + \sum _{j=0}^{t-1}\theta ^{(i)}_{j}\varepsilon ^{(i)}_{t-j} \end{aligned}$$

which represents the historical decomposition of variable *i* at time *t*. Ignoring the initial conditions,^5^ Eq. (3) can be rewritten in a matrix form as^6^:

4 $$\begin{aligned} HD_{t+i} = \sum _{j=0}^{\infty }IRF_{j}\circ \Upsilon _{t+i-j} = \sum _{j=0}^{i-1}IRF_{j}\circ \Upsilon _{t+i-j} + \sum _{j=i}^{\infty }IRF_{j}\circ \Upsilon _{t+i-j} \end{aligned}$$

where $IRF_{j}$ are orthogonalised impulse responses matrices obtained using Cholesky factorisation, $\circ$ is a Hadamard product, $\Upsilon _{t+i-j} =[\varepsilon _{t+i-j},...,\varepsilon _{t+i-j}]$ is the $n\times n$ matrix containing residuals and $HD_{t}$ is a historical decomposition matrix at time *t*. We note that the historical decomposition $HD_{t}$ in Eq. (4) is a function of impulse responses weighted by residuals $\varepsilon _{t}$. The historical decomposition is used as a standard tool for decomposing an observed variable at any given time into the model projection and the deviation from the projection due to shocks. The decomposition of Eq. (4) has two terms where the first term on the right-hand side represents the ‘base projection’ of $HD_{t+i}$ given the information available at time *t* and the second term on the right-hand side represents the difference between the actual series and the base projection due to the structural innovations in the return variables subsequent to period *t*. Particularly, it depicts that the gap between the actual series and the base projection is the sum of the weighted contributions of the innovation to particular series under consideration (Dungey et al. 2019).

The elements of $$ HD_{t,ij }$$ show the dynamic properties of the network and represent the connectedness measure for *i* to *j* denoted by $$c_{t,i \rightarrow j}$$. Thus, it is possible to analyse the connectedness matrix $$C_{t} = [HD_{t,ij}]$$ with off-diagonal elements representing the pairwise directed connectedness. Letting $$c_{t,j \rightarrow i}$$ and $$c_{t,i \rightarrow j}$$ be in-degree and out-degree, respectively (with $$c_{t,j \rightarrow i} \ne c_{t,i \rightarrow j}$$ not restricted to be identical), we can define the net-pairwise directed connectedness of *i* as $$c_{t,i} = c_{t,j \rightarrow i} - c_{t,i \rightarrow j}$$, which is not restricted to be positive.

Total directional connectedness from and to others is given by:

5 $$\begin{aligned} c_{t,i\leftarrow others}&= \sum _{i=1,j\ne i}^{n}HD_{t,ij}\nonumber \\ c_{t,others\leftarrow i}&= \sum _{i=1,j\ne i}^{n}HD_{t,ij} \end{aligned}$$

Pairwise directional connectedness for sample *n* is defined as:

6 $$\begin{aligned} c_{ij} = \frac{1}{n}\sum _{i,j=1,j\ne i}^{n}HD_{t,ij} \quad \forall i\ne j \end{aligned}$$

For the purposes of our spillover indices, this gives us the ability to propose the same form of the DY spillover index. However, it has the advantage of parameters $\theta _{i}$ not being restricted to being strictly positive, as is the case for the weights from the forecast error variance decomposition as given in Eq. (3). Consequently, we can trace a spillover or vulnerability index over time using historical decomposition, and observe not only the contributions shocks from different markets to the system but also whether these shocks amplified or dampened the transmission from the source market. The disadvantage is that our decomposition is sourced from an unconditional estimate of the system over the sample period. Thus, it does not directly capture problems that may be associated with changing underlying variance regimes in the data. This is a particularly a problem when comparing non-crisis and crisis periods. To manage this, we constructed subsample VARs for the same subsamples used in the contagion estimation. This is outlined in the following discussion on the contagion methodology so that the results are directly comparable across the two methods.

### Contagion methodology

In a latent factor model representation of the relationship between markets we might postulate that each return is exposed to both a common factor ($f_{\omega ,t}$) and an idiosyncratic factor ($f_{i,t}$) (or that it is in capital asset pricing model (CAPM) framework with a non-diversifiable and diversifiable risk). We are able to write that any individual return at time *t*, denoted $r_{i,t} \in r_{t}$

7 $$\begin{aligned} r_{i,t} = \beta _{i}f_{\omega ,t} + f_{i,t} \end{aligned}$$

where in matrix form, the system is represented by:

8 $$\begin{aligned} r_{t} = Bf_{\omega ,t} + F_{t} \end{aligned}$$

and $F_{t}$ is a diagonal matrix that represents the variances. In a CAPM framework, we invoked a market indicator or ‘mimicking factor’ to represent $f_{\omega ,t}$. This is usually in the form of market return (often an index or an equally weighted index of constituent assets). That is, the usual formulation of Eq. (7) will be:

9 $$\begin{aligned} r_{i,t} = \beta _{i}r_{o,t} + \mu _{i,t}, \ E[\mu _{i,t}] = 0, \ \text {cov}[r_{o,t},\mu _{i,t}] = 0 \end{aligned}$$

where $r_{o}$ is the asset return of possible source of contagion, $r_{i}$ is the asset return of possible target of contagion, $\beta _{i}$ is identified by the correlation between $r_{i}$ and $r_{o}$, and the idiosyncratic factors are represented by the residuals in Eq. (9).

The problem of identifying contagion arises when during different sample periods, we observed changes in the relationships between the variables, specifically changes in $\beta _{i}$ and wanted to identify the source of those changes. Consider two periods defined as period of low and high volatility—for convenience we label them *L* (low volatility) and *H* (high volatility). In the simplest case, we can observe that:

10 $$\begin{aligned}&r_{i,L} = \beta _{i,L}r_{o,L} + \mu _{i,L}, \ E[\mu _{i,L}] = 0, \quad \text {cov}[r_{o,L},\mu _{i,L}] = 0 \end{aligned}$$

11 $$\begin{aligned}&r_{i,H} = \beta _{i,H}r_{o,H} + \mu _{i,H}, \ E[\mu _{i,H}] = 0, \quad \text {cov}[r_{o,H},\mu _{i,H}] = 0 \end{aligned}$$

where $\beta _{i,L} \ne \beta _{i,H}$ and is identified by the correlation in low and high periods, respectively. The debate is then about why these parameters (or corresponding matrices for a vector of returns) have changed. Initial arguments focused on changes in volatility contributing to changes in correlation and resulting in increased non-diversifiable risk during crises due to $\beta _{H} > \beta _{L}$. Forbes and Rigobon (2002), however, demonstrated the mechanical relationship between higher volatility and higher correlation parameters. They concluded that in most cases, the increase in $\beta _{H}$ in a period of high volatility was mainly due to the interdependence of markets, rather than contagion.

Consider for example the correlation between $r_{i}$ and $r_{o}$ in the low and high periods. We know that in the simple form, we are using the correlation coefficient $\rho _{i,L}$ (low period) and $\rho _{i,H}$ (high period) that can be expressed as:

12 $$\begin{aligned} \rho _{i,L} = \beta _{i,L}\frac{\sigma _{o,L}}{\sigma _{i,L}}, \quad \rho _{i,H} = \beta _{i,H}\frac{\sigma _{o,H}}{\sigma _{i,H}} \end{aligned}$$

where $\sigma _{i,L}, \sigma _{o,L}, \sigma _{i,H}, \sigma _{o,H}$ are the volatility of returns in both the target and source markets (for both low and high periods), with a corresponding form for $\rho _{i,L}$ and $\rho _{i,H}$. Rearranging this so that parameters $\beta _{i,L}$ $\beta _{i,H}$ can be directly compared, we produced the Forbes and Rigobon (2002) result—if the increase in volatility in the source market from $\sigma _{o,L}$ to $\sigma _{o,H}$ is not exactly offset by the same rise in the volatility of the target market from $\sigma _{i,L}$ to $\sigma _{i,H}$, then the observed correlation must increase. That is, if an increase in volatility in the source market exceeds the change in volatility in the target market, we will necessarily observe $\rho _{i,H} > \rho _{i,L}$ in a way that is not consistent with contagion as an increase in the transmission of shocks in $\beta _{i}$ between the two periods. This led Forbes and Rigobon to propose a scaling adjustment to test contagion based on correlation. They concluded that most contagion identified in this manner was because of changes in underlying volatility.

The Forbes–Rigobon (FR) adjustment has been shown to under-reject the null hypothesis of no contagion (Dungey et al. 2004). This is because the change in observed volatility in the target market has two potential sources. The first is the transmission of increased volatility from the source market—that is, the increase in $\sigma _{i}$. The other is due to potential changes in volatility in the idiosyncratic component (the diversifiable risk) which is associated with the asset, which we denote $\omega _{i} = \text {var}(\mu _{i})$. Dungey and Renault (2018) provided the proof that the FR adjustment only works when idiosyncratic volatility in target markets is also unchanged between sample periods—that is, when $\omega _{i,L} = \omega _{i,H}$. Otherwise, the test on correlations will tend to over-accept the null of no contagion.

The clearest lesson from the literature on detecting contagion via changes in correlation coefficients is that although it is intuitively appealing, it is also fraught with hazard because of the number of implicit assumptions invoked. The clearest approach is to directly examine the changes in $\beta _{i}$ between periods and, at the same time, be aware that these changes have several sources of volatility influence that must be distinguished.

Consider that Eqs. (10) and (11) are our approximation of Eq. (9), where we approximate the common factor with our mimicking return, $r_{o,t}$ and that this can be represented as:

13 $$\begin{aligned} f_{\omega ,t} = br_{o,t} + \upsilon _{o,t} \end{aligned}$$

where $$\text {var}(\upsilon _{o,t+1} ) = \omega _{o}^{2}$$ and the correlation between the idiosyncratic component of $$f_{w,t}$$ and of $$ r_{i,t}$$ is denoted as:

14 $$\begin{aligned} \text {cov}(\mu _{i,t+1}, \mu _{o,t+1}) = \omega _{i,o} \end{aligned}$$

Assuming the shocks to $f_{\omega ,t}$ is independent, we find the unconditional variance of $f_{\omega ,t}$ which is not identified. The return variance of $f_{\omega ,t}$ can be extended by incorporating a constant component. This constant component represents the proportion of the factor variance explained by the mimicking return, that is:

15 $$\begin{aligned} \alpha = \frac{\text {var}(f_{\omega })}{\text {var}(r_{o,t+1})} = \frac{\sigma _{\omega }^{2}}{\sigma _{o}^{2}}, \ \alpha \in ]\ 0,1[\ \end{aligned}$$

which means that it must be large enough to capture at least part of the variation in the factor. This is done by setting a minimum value on $\alpha$ so that it must allow at least some of the variation to be captured by the common factor in all periods by setting $\alpha = \bar{\alpha }$ at the lower bound that respects this condition. We achieved this by setting $\bar{\alpha }$ as 1, minus the proportion of the unconditional variance of the mimicking asset explained by the minimum conditional variance of that asset over the sample period, that is:

16 $$\begin{aligned} \bar{\alpha } = \frac{\text {min}_{1\ll t\ll T}[\text {var}_{t}(r_{oi,t+1})] }{\text {var}(r_{oi,t+1})} \end{aligned}$$

With these definitions in mind, we can return to the form of Eq. (9) and note that:

17 $$\begin{aligned} \text {cov}(f_{i,t}, f_{\omega ,t}) = \text {cov}(r_{i,t+1},r_{o,t+1}) = b\sigma _{\omega }^{2} + \omega _{i,o} \end{aligned}$$

To obtain our expression for the components of $\beta _{i}$ (identified by the correlation between $r_{i}$ and $r_{o}$), we recognise the following:

18 $$\begin{aligned}&\beta _{i} = \frac{\text {cov}(r_{i,t+1},r_{o,t+1})}{\text {var}(r_{o,t+1})} \end{aligned}$$

19 $$\begin{aligned}&\text {var}(r_{o,t+1}) = \frac{\sigma _{\omega }^{2}}{\alpha } \end{aligned}$$

20 $$\begin{aligned}&\text {var}(r_{o,t+1}) = \frac{\omega _{o}^{2}}{1 - \alpha } \end{aligned}$$

where Eq. (18) comes from the definition of correlation, Eq. (19) comes from Eq. (15) and (20) from the definition of the variance structure of the common factor taking into account the scaling parameter $\alpha _{i}$. So, to obtain an expression for $\beta _{i}$, we scale $\text {cov}(r_{i,t+1}, r_{o,t+1} )$ by $\text {var}(r_{o,t+1}$), the second term by the equivalent value of Eq. (18), and the third term by the value Eq. (19), leaving the final expression for $\beta _{i}$ as:

21 $$\begin{aligned} \beta _{i} = \alpha _{i}b_{i} + (1 - \alpha _{i})\frac{\omega _{io}}{\omega _{o}^{2}} \end{aligned}$$

This expression shows that the parameter of interest in transmitting the shocks from the source asset to the target asset can be decomposed into two components. The first is the common transmission effect, and the second is the effect of changing conditional variances between the idiosyncratic shocks in the common and idiosyncratic factors. A test for a change in $\beta _{i}$ that does not acknowledge this may mistake changes in relative volatility for structural changes in the transmission of shocks.

We are interested in tests to detect changes in $b_{i}$ between periods. We omit, however, the source proposed by Sewraj et al. (2018), which adds a trend term specified in Eq. (10).^7^ This captures the changing integration of the target market with the source market because of increased global integration over time. We use relatively short sample periods. The evidence in Sewraj et al. (2018) suggests that the effects, while statistically significant, are economically very small (even over more than two decades of weekly data) and not evident in the crisis period.

Although we have illustrated this problem for a single asset related to a common mimicking factor, the model is easily extended to a vector of assets in relation to a single mimicking factor, and with some degree of greater complexity to the possibility of more than one mimicking factor, analogous to a multi-factor CAPM (Dungey and Renault 2018). Dungey and Renault (2018) established a method for identifying these contagion effects using conditional variance. The method is simple to use and offers insights into the source of changes in the transmission matrix over subsamples.

#### Estimation strategy

Testing for statistical changes in the parameter $b_{i}$ for assets can be achieved using generalised method of moments (GMM) and conditional second moment conditions. We know that the instrumented unconditional covariance between one asset $r_{i}$ and another $r_{j}$ (with the same mimicking portfolio asset in place for both, $r_{o}$) will be constant in our framework (Dungey and Renault (2018). However, the intuition follows from Eq. (1). This can be expressed as:

22 $$\begin{aligned} E[\ z_{t}r_{j,t+1}(r_{i,t+1} - b_{i}r_{o,t+1}) ]\ = c_{ij} \ \forall j = 0,1,..., n \ \forall i\in I_{n} \end{aligned}$$

where $z_{t}$ is a vector of instruments used to capture conditional heteroskedasticity. It is $(n+2)$-dimensional vector containing a constant and squared returns $r_{j,t}^{2}, I_{n} = 0, 1,..., n$. This implies that Eq. (22) will have unconditional moment restrictions. The moment restriction can be represented in a linear regression model as:

23 $$\begin{aligned} (r_{t+1}\otimes z_{t})r_{t+1} = b_{i}(r_{t+1}\otimes z_{t})r_{o,t+1} + [\ I_{n+1} \otimes z_{t} ]\ c_{i\bullet } + \varepsilon _{i,t+1} \end{aligned}$$

where $r_{t+1} = (r_{j,t+1})_{0\le j \le n }, I_{n+1}$ is the identity matrix of dimension $(n+1), c_{i\bullet } = (c_{ij})_{0\le j \le n }$, $\otimes$ is a Kronecker product and $\varepsilon _{i,t+1}$ is a $(n+1)(n+2)$-dimensional martingale difference sequence.

We also know that the unconditional covariance between $r_{i}$ and $r_{o}$ is constant defined by:

24 $$\begin{aligned} E[\ r_{o,t+1} ( r_{i,t+1} - \alpha _{i}b_{i}r_{o,t+1} ) ]\ = \omega _{ij} \end{aligned}$$

where $\alpha _{i}$ is to be chosen such that it is constrained by the fact that volatility must be sufficiently large to capture at least part of the variation in the factor. This assumes that a one- or two-factor model or its characterisation through moment conditions in Eqs. (22) and (24) are well-specified. Estimation of these parameters can be implemented using a GMM.^8^

These two sets of moment conditions across multiple assets are demonstrated with a single mimicking portfolio that provides sufficient identification to estimate the parameters of interest, specifically $b_{i}$ for different sample periods. We can then test the null hypothesis of $b_{i,L} = b_{i,H}$ as a more clearly specified test for the presence of contagion than of either $\beta _{i,L }=\beta _{i,H}$. This may be contaminated by changing idiosyncratic variances, or $\rho _{i,L } = \rho _{i,H}$, which may be contaminated by changes in both idiosyncratic variances and the relative variance of the assets over time.

## Empirical analysis and discussion of results

Our choice to study returns rather than volatility was guided by the literature that indicates returns have less volatile spillover effects (Yilmaz 2010). Additionally, average returns have been found to transmit most information in the Asian markets (Beirne et al. 2010).

**Table 3:** Phases in the sample

Phases	Period	Representing	Observations
Pre-GFC	1 January 2003–14 September 2008	Lead-up to the global financial crisis	1488
GFC	15 September 2008–31 March 2010	Global financial crisis	403
EDC	1 April 2010–30 December 2013	European debt crisis	979
Recent	1 January 2014–29 December 2017	Most recent period	1043

Table 3 shows the four subsample periods in our empirical analysis. The first is the pre-GFC period, from January 2003 until the bankruptcy of Lehman Brothers in mid-September 2008. The second is the GFC from then until the end of March 2010. This may be regarded as overly long compared with other analyses, and the literature is indeed mixed on whether it divides the US recovery from mid-2009 into a separate period. Dungey et al. (2015) discussed dating the crisis. The third period is the European debt crisis (EDC), which we designated as starting from the beginning of the International Monetary Fund’s program in Greece in April 2010 until the end of December 2013, at which point only Ireland and Portugal still had to finalise their recovery from the support packages implemented during the crisis. They both achieved this in 2014.^9^ The fourth period covers the most recent data from January 2014 to the end of the sample on 29 December 2017. The total number of observations in the whole sample is 3,913. Just over 30% of observations are found in the lead-up to the GFC, and approximately one quarter in each of the EDC and the post-crisis periods. The GFC period is the shortest, covering six months from the collapse of Lehman Brothers; this period contains just under 10% (403) of the total observations. Thus, each subsample has a reasonable number of observations for tractable estimation and is in line with existing demarcations of the sample periods. Table 4 shows the descriptive statistics for each equity market return for each country across the different subsamples. It is worth noting that our estimation is based on rolling window, and phases do not affect results.

**Table 4:** Descriptive statistics of each equity market return

Item	AU	CN	IN	ID	JP	HK	MY	PH	SG	KR	LK	TH	TW	US
Pre-GFC	${01.01.2003 - 14.09.2008}$													
Obs.	1488	1488	1488	1488	1488	1488	1488	1488	1488	1488	1488	1488	1488	1488
Mean	0.0004	0.0004	0.0006	0.0011	0.0011	0.0003	0.0004	0.0007	0.0005	0.0007	0.0008	0.0003	0.0005	0.0003
SD	0.009	0.0169	0.013	0.0159	0.0135	0.0125	0.0083	0.0138	0.0111	0.0139	0.0132	0.0138	0.0128	0.009
Kurtosis	5.7291	3.8583	6.8409	5.9261	4.5719	1.4816	16.8173	3.5126	2.8557	1.5977	20.948	2.5153	16.2884	2.0773
Skewness	− 0.2623	− 0.2021	0.045	− 0.7247	− 0.5222	− 0.3632	− 1.5032	0.0927	− 0.1962	− 0.2289	− 0.8049	− 0.2563	− 0.5675	− 0.0781
GFC	${15.09.2008 - 31.03.2010}$													
Obs.	403	403	403	403	403	403	403	403	403	403	403	403	403	403
Mean	0.0001	0.0012	0.0006	0.0009	0.0013	0.0001	0.0006	0.0005	0.0004	0.0006	0.0012	0.0005	0.0006	0.0001
SD	0.017	0.0203	0.0264	0.0226	0.0195	0.0241	0.0096	0.0191	0.0206	0.0214	0.0133	0.0184	0.0189	0.0231
Kurtosis	2.8761	2.3785	5.329	7.9424	5.6808	6.2907	3.5861	6.8702	2.7589	5.754	3.7389	1.9951	5.4976	4.5382
Skewness	− 0.3706	0.0451	0.4415	0.5321	− 0.3727	− 0.0805	− 0.0952	− 0.6743	0.0541	− 0.2037	0.3388	− 0.0536	− 0.7909	0.0471
EDC	${01.04.2010 - 30.12.2013}$													
Obs.	979	979	979	979	979	979	979	979	979	979	979	979	979	979
Mean	0.0001	-0.0003	0.0002	0.0002	0.0005	0.0005	0.0004	0.0006	0.0001	0.0002	0.0005	0.0001	0.0006	0.0005
SD	0.0095	0.0117	0.0118	0.0105	0.0123	0.0137	0.0058	0.0122	0.0089	0.0118	0.0088	0.0107	0.0116	0.0106
Kurtosis	1.4118	2.1793	2.7072	0.7026	6.1232	5.3418	4.3511	4.1581	1.7707	3.3208	4.1259	2.0014	3.3968	4.4625
Skewness	− 0.1701	− 0.2237	− 0.1805	− 0.0335	− 0.5283	− 0.7564	− 0.4458	− 0.4674	− 0.3717	− 0.2069	0.2883	− 0.161	− 0.1546	− 0.3514
Recent	${01.01.2014 - 29.12.2017}$													
Obs.	1043	1043	1043	1043	1043	1043	1043	1043	1043	1043	1043	1043	1043	1043
Mean	0.0002	0.0005	0.0003	0.0006	0.0004	0.0004	0	0.0004	0.0001	0.0002	0.0001	0.0003	0.0003	0.0004
SD	0.0082	0.015	0.0102	0.0084	0.0083	0.0127	0.0048	0.0094	0.0073	0.0073	0.0047	0.0086	0.0075	0.0075
Kurtosis	1.765	7.446	2.9552	4.4753	3.7315	5.9324	3.9838	3.9585	2.9142	1.714	4.3	2.8796	6.2104	3.2866
Skewness	− 0.278	− 1.1872	− 0.2879	− 0.7474	− 0.3159	-0.0207	− 0.5252	− 0.4318	− 0.1487	− 0.2335	− 0.382	− 0.1661	− 0.4943	− 0.3544

### Evidence for spillovers

Table 5 shows the average spillovers obtained from historical decomposition of shocks to the observed returns of each country in the sample for the whole period. The rows represent the recipient markets for shocks spreading from source countries which are shown in each column. The shocks have different magnitude and are distinguished by sign. Negative numbers represent a reduction in returns as a result of the shock; positive shocks represent an increase in returns.

**Table 5:**
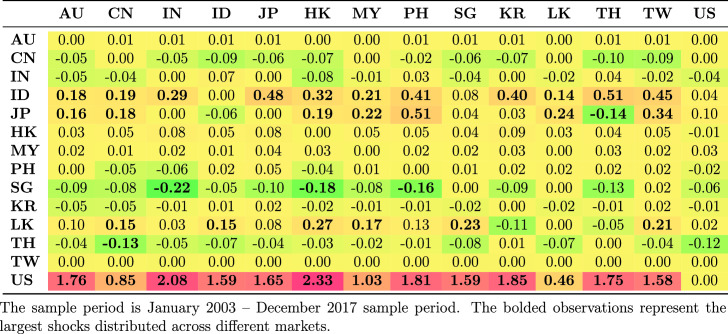
Spillovers for the entire period

The US receives more shocks than it transmits. This is common because each market is exposed to shocks from many markets and distributes its own shocks to many markets. The US receives positive shocks from Asian countries, on average increasing its return, while it also transmits shocks, though with less magnitude, to Asian countries. These outcomes are generally consistent with the US being the safe haven market when international stress occurs. The US markets benefit from flight to safety and familiarity, and benefit from the hypothesis of Kaminsky and Reinhart (2003) that the US operates as a central market redistributing shocks received from peripheral markets to other markets. This implies that the are other channels through which vulnerability spread from the US during the GFC to other markets apart from the stock market. Credit default swap is the dominant channel through which shocks spread from the US to small Asian economies. Other channels include trade linkages through the fluctuating exchange rates and interest rates.

Unlike the US, which receives positive shocks, China receives negative shocks from most other markets, although the magnitude of these shocks is low. Indonesia and Japan receive the largest positive shocks from other Asian markets, but transmit smaller shocks to other Asian markets.

**Table 6:**
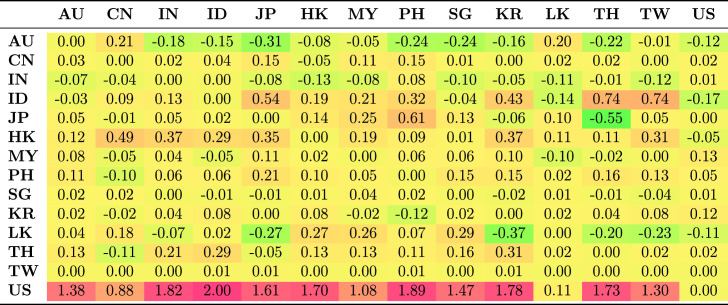
Spillovers for the pre-global financial crisis sample period (2003–2008)

**Table 7:**
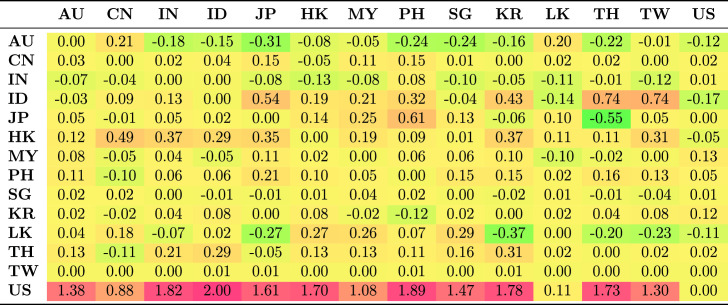
Spillovers for the global financial crisis sample period (2008–2010)

**Table 8:**
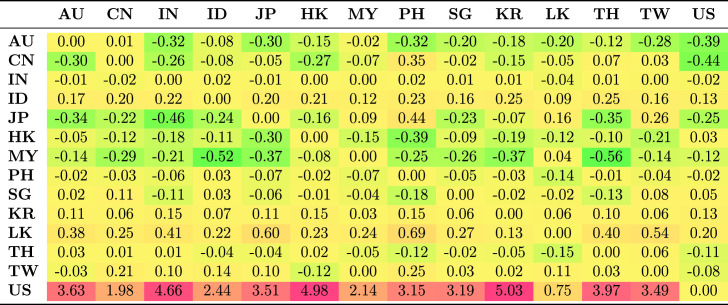
Spillovers for the European debt crisis sample period (2010–2013)

**Table 9:**
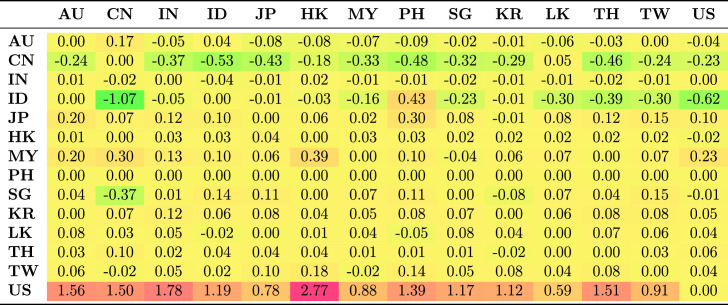
Spillovers for the most recent sample period (2013–2017)

Further, we considered how the transmission of shocks changes over time by examining the four periods. The results in Tables 6, 7, 8 and 9 clearly show that the transmission of shocks from different markets changes in each phase. During the pre-GFC period, the US became the recipient of larger positive shocks from Asian markets compared to the GFC period. The US also transmitted more shocks to Asian markets than it absorbed in the GFC period. The magnitude of shocks it received dropped in the GFC period compared with the pre-GFC period. This suggests that Asian markets were less involved in spreading shocks to the US during the GFC period.

**Fig. 2:**
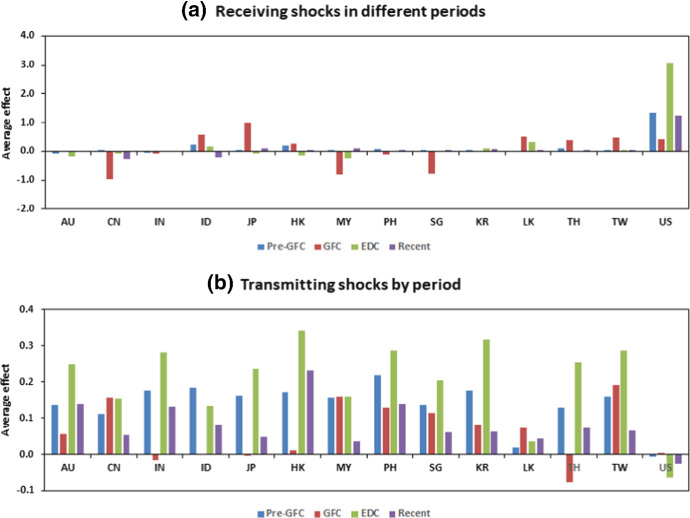
Average shocks received and transmitted by period and market. The sample period is January 2003–December 2017. The source of the data is Thomson Reuters Datastream

Figure 2a shows the estimated absorption of shocks by a market, while Figure 2b shows the transmission of shocks from a market. The spillover effect for each market during each phase is given in separate columns. Figure 2b clearly shows that in the pre-GFC period, the average spillover effect transmitted by the market to others in the system was roughly similar, mainly in the range of 0.1–0.2 with the exceptions of an almost neutral transmission from Sri Lanka and the US. The average effect ($-0.0063$) was negative and very small in the US.

Compared with the European debt crisis (EDC) and the current periods, the extent of the shocks during the pre-GFC period was small (see Tables 6, 7, 8 and 9).

Australia and India were among the countries to receive, on average, negative effects on their returns as spillovers from the rest of the markets. Indonesia, Hong Kong and Thailand received return-enhancing spillovers. The other markets fell between these two alternatives, although the range is not high.

During the GFC period, the transmission of shocks from the Asian markets generally declined compared to the pre-GFC period. While there is some evidence that the transmission of these shocks increased returns in other markets via spillovers, there is even less evidence that they reduced returns, except for spillovers from Thailand. Table 7 shows this is mainly through spillovers with China, Malaysia and Singapore. Spillover effects from shocks received during the GFC period vastly decreased from the pre-GFC period. Most sample markets continued to receive, on average, the same sign effect of shocks in both periods, although Malaysia and China received opposite average effects. For Japan, these were spillovers that increased returns, which is consistent with the flight to quality, safety, and familiarity in the region. The spillover effects for China were strongly negative, reflecting the expected decline in the country’s economic expansion in response to a weaker global economy. Malaysia and Singapore, also open and export-dependent economies, experienced negative spillovers in the GFC period. The US gets some positive spillovers because of the flight to safety and leverage effects. South Korea experienced relatively little change, with the average effect of spillovers that remaining neutral in both periods.

The EDC period contrasts strongly to the pre-GFC and GFC periods, with the scale of spillovers into and out of markets being similar; almost all markets experienced positive spillovers (see Table 8). That is, spillovers resulted in higher returns in these markets, and spillovers from Asian markets resulted in higher returns elsewhere. This may reflect that the crisis originated in Europe and the debt markets of Asia were perceived as more robust, thereby providing an alternative investment opportunity during the EDC period.^10^ In contrast, spillovers to and from the US were negative. In other words, spillovers from the USA reduced returns in Asia, reflecting uncertainty in world markets, and spillovers from Asia reduced returns in the US.

The most recent period shows a return to conditions more similar to the pre-GFC period in terms of transmission effects. These were, if anything, slightly smaller than in the other periods, but produced positive returns in Asian markets. The exception again was the US, where the out-coming spillovers tended to reduce returns in other markets with a larger effect than in the pre-GFC period of -0.0275.

Table 9 shows that transmissions to Indonesia and China were important components of this overall result. In contrast, the external spillovers that other markets received in recent times generally had little effect on returns. The scale of shocks to the US was considerably larger than for other markets, and these effects were positive, implying that spillovers from other markets, on average, increase US returns. Most markets received negligible spillovers from others. The exceptions were Indonesia, China and the US. Indonesia and China seemed to be intertwined in a form of feedback in which spillovers between them (see Table 9) mutually reinforce lower returns.^11^ The spillover effects on the US were substantially larger than in the other periods, and primarily reflected combinations of Indonesian and Chinese spillovers, although offsets from Malaysia also played a role.

The different roles that China and the US played in the transmission mechanism to and from Asian markets are evident in this analysis, and because of this, we examined more closely the spillovers originating from these markets. Table 10 shows the total contributions of spillovers to and from China and the US on other markets over the four periods. This allowed for a preliminary analysis of the extent of change between the transmissions between these markets before formal tests for contagion are conducted (see Sect. 5.2).

**Table 10:** Summary of the spillovers from and to China and the US by other markets

From	To	Pre-GFC	GFC	EDC	Recent	From	To	Pre-GFC	GFC	EDC	Recent
*Panel A: from China to others*						*Panel B: from the US to others*					
CN	AU	0.2100	− 0.0252	0.0130	0.1705	US	AU	− 0.1190	− 0.0318	− 0.3942	− 0.0372
	IN	− 0.0411	− 0.2200	− 0.0195	− 0.0150		CN	0.0167	− 0.8390	− 0.4409	− 0.2254
	ID	0.0943	0.3970	0.1987	− 1.0677		IN	0.0128	− 0.2100	− 0.0172	0.0039
	JP	− 0.0059	2.1835	− 0.2179	0.0663		ID	− 0.1680	0.6440	0.1285	− 0.6229
	HK	0.4910	0.0427	− 0.1151	0.0033		JP	0.0035	1.2752	− 0.2536	0.1028
	MY	− 0.0466	− 0.4780	− 0.2939	0.2970		HK	− 0.0542	0.0369	0.0331	− 0.0182
	PH	-0.0984	-0.0197	-0.0321	0.0005		MY	0.1310	− 1.0102	− 0.1155	0.2344
	SG	0.0193	− 0.2490	0.1073	− 0.3653		PH	0.0536	− 0.1930	− 0.0192	0.0002
	KR	− 0.0233	− 0.1150	0.0635	0.0738		SG	0.0086	− 0.3690	0.0488	− 0.0076
	LK	0.1790	− 0.0625	0.2525	0.0306		KR	0.1150	− 0.2410	0.1321	0.0473
	TH	− 0.1110	− 0.0084	0.0124	0.1007		LK	− 0.1090	0.1060	0.2042	0.0357
	TW	0.0025	0.5500	0.2142	− 0.0228		TH	0.0233	0.5180	− 0.1060	0.0638
	US	0.8770	0.1790	1.9786	1.4964		TW	− 0.0026	0.3250	− 0.0788	0.0382
*Panel C: from others to China*						*Panel D: from others to the US*					
AU	CN	0.0307	− 1.4987	− 0.2981	− 0.2408	AU	US	1.3848	0.602	3.6317	1.5591
IN		0.0182	− 1.4184	− 0.2555	− 0.3695	CN		0.8770	0.1790	1.9786	1.4964
ID		0.0385	− 1.3310	− 0.0783	− 0.5253	IN		1.8162	0.6210	4.6569	1.7765
JP		0.1510	− 1.2764	− 0.0507	− 0.4304	ID		2.002	0.4400	2.4422	1.1887
HK		− 0.0477	− 1.8043	− 0.2706	− 0.1757	JP		1.6059	0.4740	3.5074	0.7753
MY		0.1130	− 0.0597	− 0.0656	− 0.3278	HK		1.6958	0.7460	4.9758	2.7652
PH		0.1540	0.5190	0.3476	− 0.4781	MY		1.0832	0.2560	2.1446	0.8784
SG		0.0106	− 1.1891	− 0.0217	− 0.3172	PH		1.8899	0.5330	3.1454	1.3929
KR		− 0.0013	− 0.9630	− 0.1451	− 0.2927	SG		1.4653	0.5180	3.1904	1.1747
LK		0.0162	− 1.0169	− 0.0465	0.0499	KR		1.7828	0.4300	5.0325	1.1225
TH		0.0190	− 1.1765	0.0658	− 0.4586	LK		0.105	0.2200	0.7506	0.5898
TW		− 0.0046	− 1.3771	0.0309	− 0.2443	TH		1.7334	0.3970	3.9693	1.5098
US		0.0167	− 0.8390	− 0.4409	− 0.2254	TW		1.3014	0.5290	3.4928	0.9088

**Fig. 3:**
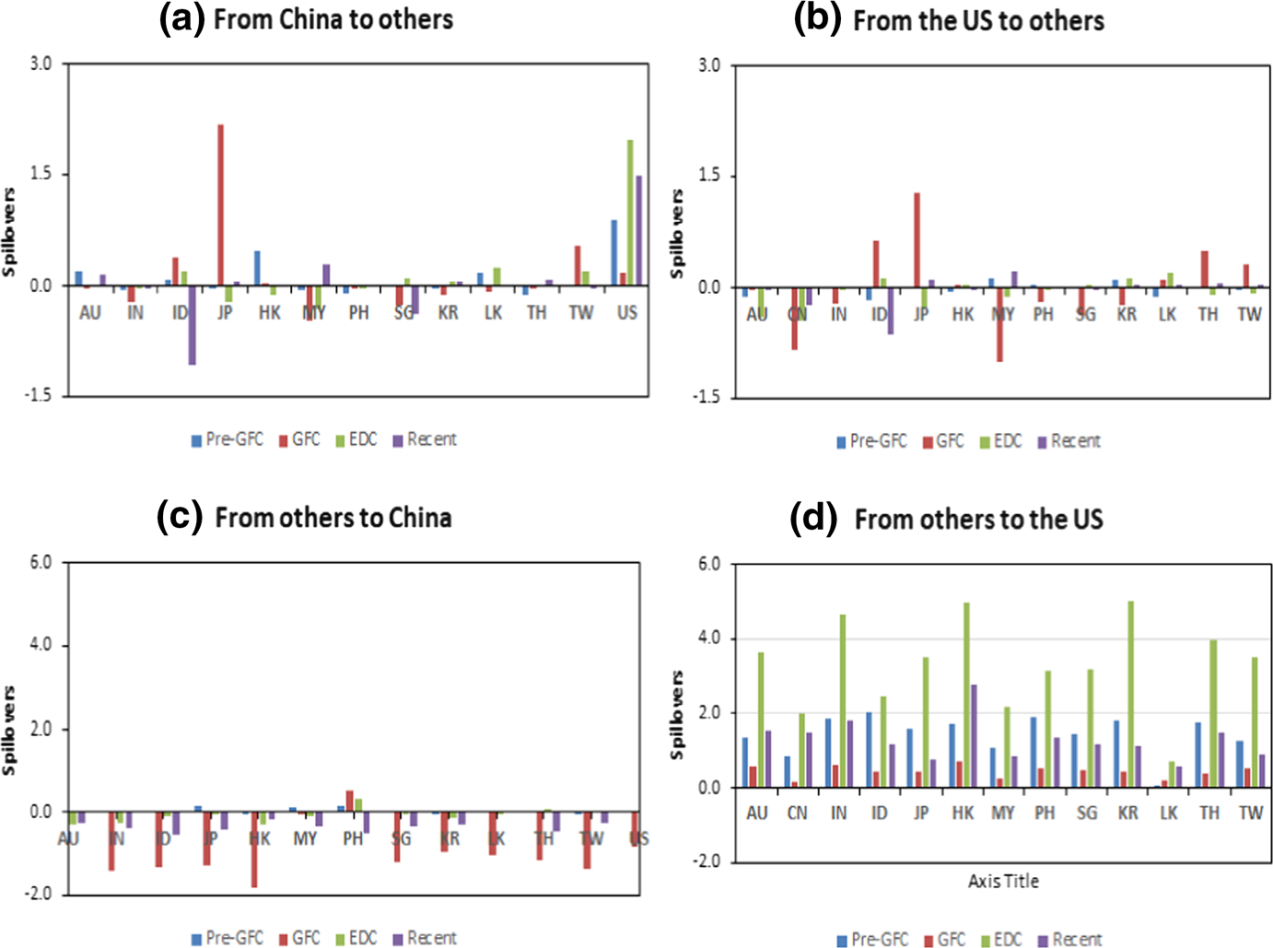
Spillovers to and from the USA and China

The results of Table 10 are plotted in Fig. 3. The scales in Fig. 33a, b for the transmission of spillovers are substantially smaller than those for receiving spillovers, as explained previously. The transmissions in Fig. 33a, b show that spillovers from China and the US were larger in the GFC period than in other periods. In both cases, the largest spillovers during the GFC period from both these sources were to Japan, indicating its importance in the region. During the EDC period, spillovers were calmer, although there is evidence that some began to, on average, change direction. Thus, Malaysia, Hong Kong and Japan, for example, demonstrated the opposite total spillover effect in this period comparing the GFC period.

The analysis of spillovers from other markets to China and US in Fig. 33c, d shows stark differences in scale and direction. The spillovers to China from other markets were predominantly negative, particularly during the GFC, but were smaller in magnitude comparing to the US. The spillovers received by the US were positive for each of the four periods (this was an average effect for the period) and were greatest during the European debt crisis period. The spillovers to the US reduced but remained positive during the GFC, compared with the pre-GFC period for many markets implying reduced attractiveness of the US markets during this crisis. During the EDC, when the US assets became much more attractive than those of Europe (hit by European debt crisis), spillovers to the US from Asian markets increased substantially. In the most recent period, the extent of average spillovers reduced, but remained higher than in the pre-GFC period.

The clearest result from the analysis of Table 10 and Fig. 3 is that spillovers from China to the US were negative but shrinking across the four periods, while spillovers from the US to China were positive and arguably growing. This is consistent with the narrative that the US and China are becoming more internationally intertwined, and that improvements in both economies can be expected to flow through to each other. In the most recent period, there is less evidence of fear of China’s spillovers having negative implications for the US economy, pointing to a more developed market relationship. Arslanalp et al. (2016) showed that the effect of shocks from China on the US is increasing.

**Fig. 4:**
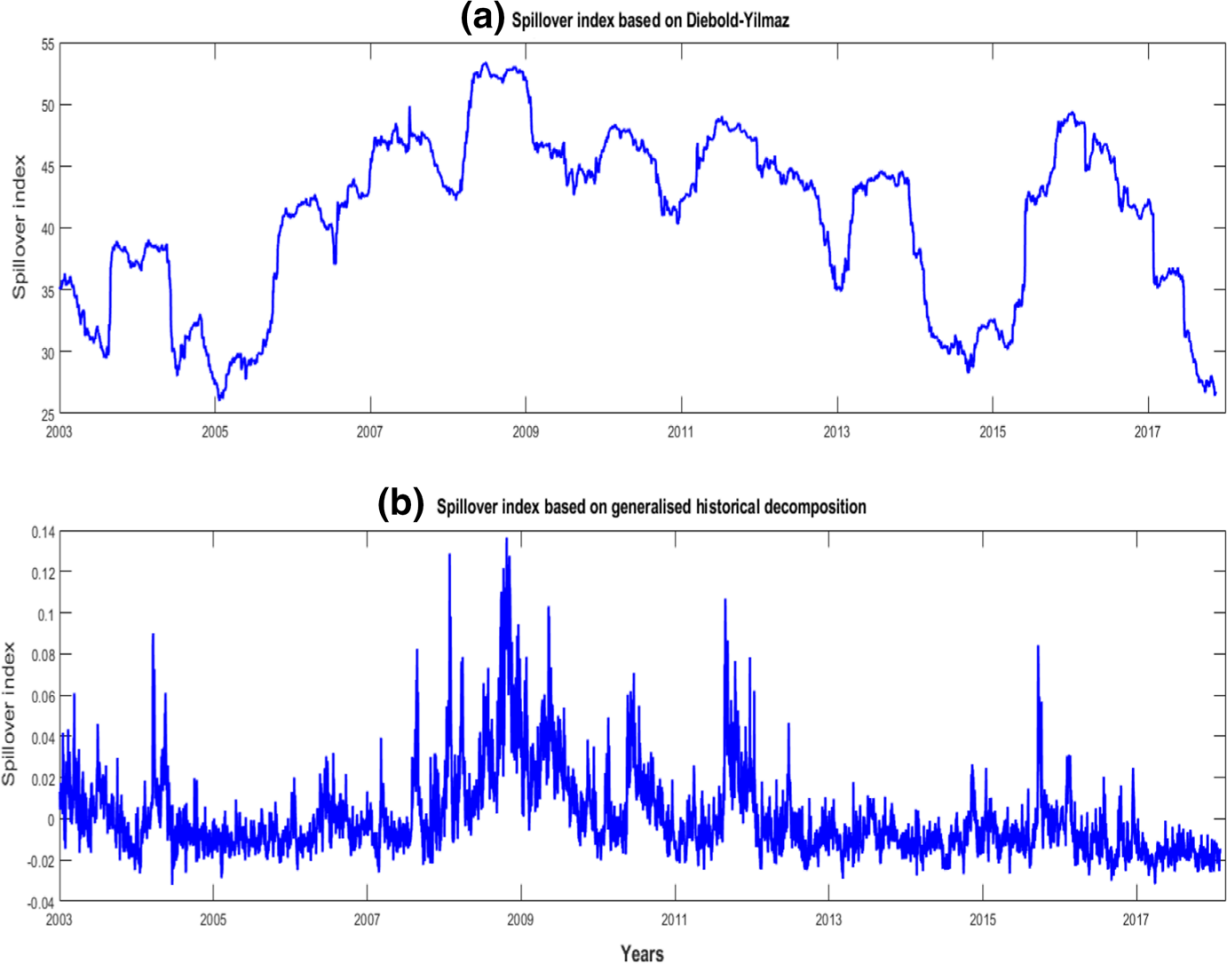
Spillovers index based on Diebold–Yilmaz and generalised historical decomposition

We next estimate the time varying spillover index based on both the DY and GHD index. Figure 4a shows the DY spillover index for the network of returns produced using a 200-day moving window. As the corresponding generalised historical decomposition (GHD) figure for returns is uninformative, we instead provide the GHD for the volatility network in Fig. 4b.

The results show that the spillover index for the entire network ranged from 30 to 50% over the 2003–2017 sample period, beginning and ending near the minimum of the range. The DY spillover index shows a substantial increase in spillovers between markets from 2005. This peaked in the second half of 2008, and is consistent with the timing of the collapse of Lehman Brothers and the associated turmoil. The index calmed somewhat after the GFC period, with an increase in spillovers associated with the EDC. In the most recent period, however, the index fell in 2014, rose over 2015, and dropped rapidly in 2017. A prominent feature of the index is the role of the choice of window length. Here, sensitivity to the choice is readily apparent in Fig. 4a, as critical observations drop in and out of the rolling sample.

The GHD spillover index in Fig. 4b shows distinct periods in which transmissions contributed to higher or lower volatility in the entire financial system. Observations below the zero line indicate cases in which transmissions in the network dampened volatility, that is, the network was robust in the sense that shocks were dampened by its structure. Positive observations indicate instances in which the network’s structure amplified the effects of the shocks. Figure 4b shows that from mid-2004 to mid-2007, the network primarily dampened the shocks, that is, it displayed a robust structure. There was a slight period of amplification in late 2006, but this is dwarfed by subsequent high-amplification effects in the network from mid-2007 to mid-2009. These are the largest absolute values in Fig. 4b and indicate that shocks during this period caused a substantial amplification in the network’s volatility transmission. The network became fragile in line with Acemoglu et al. (2015) and Haldane (2009). The results concur with the analysis of Dungey et al. (2019c), in which the fragility of a network of global sovereign and financial institution CDS increased to the stage that almost the entire network can be expected to default in response to a tail shock. The GHD spillover index shows that the amplification effect calmed somewhat in 2009, before flaring again during the Greek debt crisis in 2010 and the European debt crisis in 2011–2012.

From late 2012 to 2015, the network returned to a more robust structure, in which its effects dampened the impact of shocks. Some abrupt interruptions to the GHD spillover index during 2015–2016 indicate short, sharp periods of amplification in the network. These are linked to China; for example, August 2016 witnessed changes to the exchange rate regime and 8% was wiped from the value of the country’s stock market on Black Monday. Arslanalp et al. (2016) documented the extreme movements in the Chinese equity market and examined the strong co-movement of Asian markets with China on 11 August 2015 and 4 January 2016. Global markets were rocked again by the unexpected outcome of a June 2016 vote in the UK to leave the European Union and the subsequent political turmoil across global markets. Although political uncertainty continued to affect major markets for the rest of 2016, it did not trigger the same level of network fragility. The network was robust again by 2017, when shocks were no longer amplified by the network structure.

### Evidence for contagion

Given the dominant role of transmissions from China and the USA in our analysis of spillovers, we now explore the more abrupt changes in transmission by examining the evidence for contagion across these markets and subsamples. For completeness, we provide the results of the uncorrected and Forbes and Rigobon (2002) corrected contagion tests for each period preceding the subsequent period—that is, whether there is contagion (a statistically significant rise in correlation), interdependence (no significant change) or decoupling (a statistically significant fall in correlation) from one period to the next.^12^ Table 11 shows the results for transmissions from China and US as source markets for each period. The usual Forbes–Rigobon (FR) style results are evident; without the correction for changing variance, the correlation tests reject the null of no contagion almost always. After the correction, the prevailing evidence is for interdependence or decoupling. The original FR approach did not test for decoupling; instead, only a one-sided test was done to detect a rise in correlation as contagion. Later research extended this to two-sided tests and, more recently, Caporin et al. (2018) labelled the reduced correlation outcome as decoupling. Table 11 shows the difficulty of reconciling the evidence from different contagion-based tests. Tests must be conducted with a thorough understanding of the compromises made in the procedure to achieve identification and empirical tractability. The arguments presented in this paper’ (see Sect. 4.2) examined the reasons for preferring the approach in Dungey and Renault (2018) to use conditional correlations rather than those based on unconditional correlations from Forbes and Rigobon (2002), both with and without corrections.

**Table 11:** Contagion results for China and the USA using the Forbes–Rigobon uncorrected and corrected tests and the Dungey–Renault test

	Originating with the USA									Originating with China								
	Pre-GFC to GFC			GFC to EDC			EDC to recent			Pre-GFC to GFC			GFC to EDC			EDC to recent		
AU	D	I	D	C	C	D	D	I	D	**C**	**C**	**C**	**C**	**C**	**C**	**D**	**D**	**D**
CN	I	I	C	I	C	C	I	C	C	–	–	–	–	–	–	–	–	–
IN	D	I	D	I	C	D	I	C	D	**C**	**C**	**C**	I	C	C	I	D	D
JP	D	I	D	I	C	D	I	I	D	C	C	D	I	C	C	**D**	**D**	**D**
HK	I	I	D	I	C	D	I	C	C	C	C	D	I	C	C	**D**	**D**	**D**
MY	D	I	D	D	I	D	I	C	D	C	C	D	I	C	C	I	D	D
PH	D	I	D	I	I	D	C	C	D	C	I	D	I	C	C	**D**	**D**	**D**
SG	I	I	D	I	I	D	I	I	D	**C**	**C**	**C**	D	I	D	I	I	D
KR	D	I	D	I	C	C	D	I	D	C	C	D	I	C	C	**D**	**D**	**D**
LK	D	I	C	I	C	C	I	I	D	C	C	D	I	C	C	**D**	**D**	**D**
TH	I	I	D	I	I	D	I	I	C	I	I	C	I	I	C	C	I	D
TW	D	I	D	D	I	D	I	C	D	C	C	D	I	C	C	**D**	**D**	**D**
US	–	–	–	–	–	–	–	–	–	I	I	D	I	C	C	I	I	C

Bold represents the scenario in which all the contagion tests results come to the same conclusion

*C* Contagion, *D* decoupling, *I* interdependence, *DR* Dungey–Renault, *FRU* Forbes–Rigobon uncorrected, *FRC* Forbes–Rigobon corrected

– Represent no detection to itself

Table 12 presents the evidence for contagion from the conditional correlation tests of Dungey and Renault (2018) using the US market as the mimicking factor during each period. We conducted a Ghysels-Hall test for structural change between the adjacent periods and a Hall test for the stability of parameters between the periods. The individual results are not reported because, in each case, the null of no change was rejected at standard significance levels.

**Table 12:** Estimates of $b_{i}$ for each subperiod with mimicking factor given by the US market

Market	Pre-GFC	GFC	EDC	Recent
AU	2.066	1.402	1.483	0.173
CN	0.485	1.209	0.786	3.053
IN	3.817	0.866	1.055	0.759
ID	4.416	1.133	1.618	0.102
JP	3.664	1.195	1.072	2.06
HK	2.965	1.759	1.944	1.095
MY	4.094	0.650	1.323	0.250
PH	4.068	1.674	1.759	0.578
SG	3.750	0.609	1.488	0.258
KR	5.129	0.927	2.620	0.372
LK	− 0.500	0.747	0.275	0.609
TH	3.044	0.130	1.795	0.497
TW	3.964	0.961	1.601	0.145

In each case, estimates are statistically significant at a 1% level and are statistically different for each market between periods

**Fig. 5:**
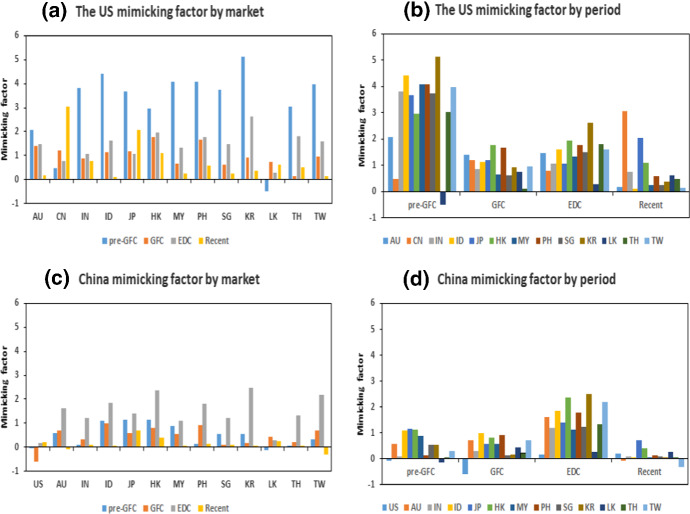
Structural transmission parameters to and from the US and China

Figure 5 provides the estimated $b_{i}$ parameter by market and sample period. It is clear from Fig. 5a, b that the loading on the mimicking factor in the pre-crisis period is generally greater than at any other part of the sample period. For most markets, the part of the relationship that is stable and not dependent on the relative volatilities of the individual and mimicking markets is higher in the pre-GFC period, and lower in other periods. In fact, for nine of the 12 markets, the value of the $b_{i}$ parameter dropped markedly from the pre-GFC to the GFC period and increased again (though only slightly) in the EDC before falling in the most recent period. Consequently, we observed a decoupling of these markets from the US market over the four periods. From the GFC period to the EDC, there is some evidence of recoupling (after the GFC), but this is limited and short-lived in size compared with the extent of the decoupling. This is consistent with Kim et al. (2015), who found that the contagion effect of the US financial crisis on Asian economies was detectable but short-lived.

A few other countries—Japan, China, Sri Lanka and Thailand—displayed different patterns in their relationship with the US mimicking factor. Sri Lanka was the only market to show a negative relationship with the mimicking factor in the pre-GFC period and in the sample as a whole. This could relate to the Sri Lankan civil war occurring at that time effectively outweighing external financial events. The occurrence of the GFC period resulted in a substantial increase in the estimated $b_{i}$ parameter for Sri Lanka indicates substantial contagion. From the GFC, however, the relationship between the Sri Lankan market and the US mimicking factor returned to the steady decoupling pattern observed with most of the other markets. Thailand differed from the other markets in that it experienced a substantial decoupling from the pre-GFC to the GFC period. After recoupling during the European debt crisis period, Thailand decoupled but remained more connected to the US mimicking factor than it was during the GFC period. This is unusual relative to the other markets.

In the Japanese case, the market decoupled from the US mimicking factor during the GFC and the European debt crisis periods, which is consistent with the resilience of Japanese markets during these periods of stress.^13^ In the most recent period, however, Japan recoupled with the US market. This relationship is not as strong as it was in the pre-GFC period, but is more pronounced than in the intervening periods and it has the second highest parameter value for the most recent period. China had the largest relationship with the US mimicking factor in the most recent period. Unlike the other markets, the relationship between China and the US markets increased over the entire sample period, albeit with a slight disruption during the EDC. That is, a formal test for contagion identified an increased correlation between the pre-GFC and GFC periods, and the EDC and most recent periods, both of which are consistent with contagion. China became more sensitive to shocks emanating from the US mimicking factor in the most recent period.

The analysis so far is consistent with the emerging importance of China as a major financial market for Asia. Due to the increasing influence of China, we now consider the test results when using the country as the mimicking factor of world conditions. In other words, what evidence is there of contagion from market conditions to other Asian countries when China represents the behaviour of the global factor? The resulting $b_{i}$ parameter estimates are shown in Table 13 and Fig. 5c, d. The results show that using China as the mimicking factor does not result in loadings that are as large as when using the US as the mimicking factor. This is not surprising given the role of the US in the world, and it indicates that the country is a better indicator of the common conditions faced by these markets. This is consistent with much of the literature. However, it also indicates that the nature of the relationship with the mimicking factor defined by China market has altered over time (Yilmaz 2010).

**Table 13:** Estimates of $b_{i}$ for each subperiod with the mimicking factor of the Chinese market

Market	Pre-GFC	GFC	EDC	Recent
AU	0.583	0.712	1.624	− 0.093
IN	0.105	0.314	1.208	0.107
ID	1.108	0.979	1.860	0.047
JP	1.148	0.584	1.409	0.711
HK	1.140	0.815	2.383	0.413
MY	0.900	0.564	1.116	0.045
PH	0.124	0.936	1.795	0.126
SG	0.547	0.115	1.227	0.091
KR	0.532	0.163	2.498	0.060
LK	− 0.140	0.430	0.271	0.266
TH	0.057	0.220	1.340	0.069
TW	0.309	0.711	2.200	− 0.307
US	− 0.061	− 0.595	0.177	0.203

In each case, estimates are statistically significant at a 1% level and are statistically different for each market between periods

The relationship of most of the 12 countries with the China mimicking factor was highest during the EDC; this is consistent with evidence of contagion represented by a significant change in the $b_{i}$ parameter from the GFC period to this period emanating from China market. The interesting aspect of this is that the increase in correlation was not necessarily a ‘bad’ outcome for many markets. This provided an avenue of alternative financial leadership and investment opportunities during a period of turmoil in developed markets. As far as we are aware, this feature has not been noted before. Here, we have an instance in which the propagation of shocks from one market source (with China as the mimicking factor) to individual markets increased in a statistically significant way. This is consistent with the definition of contagion but would not be viewed as necessarily harmful in this application.

We now explore the possibility that the Chinese market does not mimic the crisis-originating part of the market but should instead be considered a diversification opportunity. Here, there are two potentially offsetting effects: a turmoil factor for developed markets represented by the US market and an opportunistic alternative for investment funds in the Asian region. This may represent a market that is better understood as having two countering forces. A similar argument has been mounted for the role of Greece and Germany in the European debt crisis, where Greece represents the problem of the crisis countries and Germany the countries that experienced demand via flight to quality (Caporin et al. 2018; Dungey and Renault 2018). A similar situation occurred when Mexico joined the North American Free Trade Agreement. Rigobon (2002) noted that Mexico’s market changed from being clearly aligned with Latin American markets to behaving more in line with North American markets.

To examine this hypothesis more closely, we specified the conditional correlation model to consider the possibility of two distinct sources of market information, with China and the US markets providing the mimicking factors. This represents a generalisation of the model given for contagion in the discussion on detecting contagion and vulnerability in Eq. (7), where:

25 $$\begin{aligned} r_{it} = \beta _{i,1}f_{\omega 1,t} + \beta _{i,2}f_{\omega 2,t} + f_{\omega i,t} \end{aligned}$$

The two common factors and the associated propagation parameters can be expressed as:

26 $$\begin{aligned}&\beta _{i1} = \alpha _{1}b_{i,1} + (1 - \alpha _{1} ) \frac{\omega _{i,o1}}{\omega _{o,1}^{2}} \end{aligned}$$

27 $$\begin{aligned}&\beta _{i2} = \alpha _{2}b_{i,2} + (1 - \alpha _{2} ) \frac{\omega _{i,o2}}{\omega _{o,2}^{2}} \end{aligned}$$

The tests of interest are the stability of the parameters $b_{i1}$ and $b_{i2}$ over the different subsamples, in which both are estimated in a joint specification.^14^ This specification has the distinct advantage of dealing with multiple sources of contagion simultaneously, which is not typically accessible in the standard FR correlation tests, though it can be encompassed in other approaches. When using this model, we found the parameterisation was not supported by the data. The independence of the two factors is compromised in the specification because China’s returns are themselves subject to large effects from the US. Therefore, we conclude that the two-factor specification based on China and the US as the two mimicking factors is not sufficiently empirically supported in the data.

### Comparison of spillovers during the GFC and COVID-19 pandemic

The global COVID-19 pandemic has severely disrupted financial markets and the real economy across the world. Due to these disruptions in the global markets, we compare the shock transmissions between the GFC and the COVID-19 pandemic periods. We investigate whether the transmission channels are the same. Table 14 shows the transmission of shocks during the pandemic. Unlike Table 7, where the Asian economies were less involved in spreading shocks to the US, the COVID-19 pandemic results show the Asian markets becoming more involved in the transmission of shocks to the US. The results suggest that the transmissions of shocks during the GFC and the COVID-19 pandemic were different.

**Table 14:**
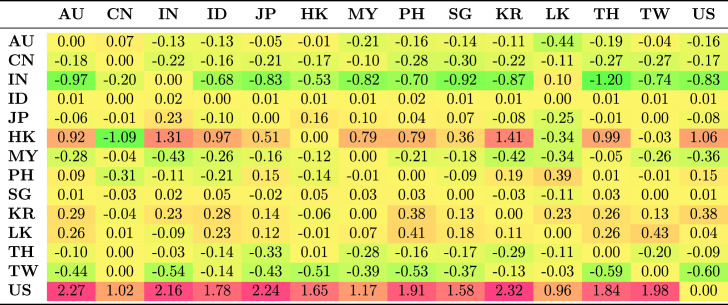
Spillovers for the global COVID-19 pandemic sample period (January 2020–August 2022)

## Implications of results

Several proposals have been made to identify the driving forces of changing financial market networks. The most common are trade and financial linkages, primarily through international banking, private and public debt ownership and related areas. There is evidence that growing international trade is associated with increasing financial integration. Elekdag et al. (2012) and Aizenman et al. (2015), for example, both used a CAPM framework to show how the estimated beta of Asian markets is increasing, and that the increase is positively associated with growing trade. Arslanalp et al. (2016) reported that increasing spillovers from China to other Asian markets are related to trade linkages. However, Avdjiev et al. (2019) showed that trade effects can be offset by the impact of financial flows in their study on the impact of dollar’s appreciation on emerging market capital flows. An appreciating dollar results in lower cross-border bank flows for emerging economies that despite improved export prospects, the portfolio channel of transmission can dominate to the extent that it worsens economic growth prospects. Thus, the foundations of the trade channel of transmission are more complex than they first appear, and it is not clear that equity market spillovers can be expected to mirror trade spillovers.

Recent research has investigated the effects of cooperation versus self-directed policy outcomes. These coordination effects have been found to be small in the monetary policy literature. Agénor et al. (2017), however, applied a similar approach to macro-prudential policies. They constructed a stylised dynamic stochastic general equilibrium model to examine how spillovers in financial markets can affect countries experiencing financial frictions. The model was calibrated to consider the problem of the benefits of coordination between emerging and advanced economies when viewed through a core-periphery lens. They found that substantial gains can come from coordinating macro-prudential policy responses across countries; however, these gains are correlated with both the size of the economies and the degree of financial friction.

We considered the simple correlation of our spillover results with trade measured as the average annual trade volume in dollars (from United Nation Comtrade statistics) and to the size of an economy, using gross domestic product (GDP) per capita. We identified the correlation between incoming spillovers and GDP per capita as positive at 0.1335. However, GDP per capita and outward spillovers were correlated at − 0.0170. That is, as an economy increases in size, the spillovers it transmits have a progressively more dampening effect on other markets. This aligns with the centre and periphery style of analyses, in which larger core developed markets receive more shocks than do perpetrators (Kaminsky and Reinhart (2002)), although we emphasise that these results are weak. We also consider the relationship of GDP per capita to absolute spillovers (|absorption| + |Transmissions|) and identified a correlation of 0.1728. Thus, our evidence only slightly supports the hypothesis in Agénor et al. (2017)—that spillovers and the size of an economy are positively related. The correlation of the different spillover measures with trade measured as either imports, exports, the sum of imports and exports, and net trade show that receiving spillovers is correlated with imports. Here, the correlation coefficient is 0.4021, which is more than the correlation of exports with outward spillovers at − 0.1880. The sum of absolute spillovers transmitted and received was also positively related to the sum of exports and imports (or the openness of an economy) at 0.3960 in our sample. These results attest to the difficulties in directly relating spillovers to trade, particularly for exports.

Agénor et al. (2017) showed that the distribution of gains from macro-prudential coordination is distorted towards larger emerging market economies and away from core economies. This is likely to cause political tensions in trying to coordinate with smaller emerging markets that end up benefiting less than larger emerging markets, and where most of the transfer will come from advanced economies. Further, obtaining redistributions from emerging markets, even when they can be demonstrated to be welfare-improving at the global level, may be politically contentious. It is worth noting that the Agénor et al. (2017) model has limitations and simplifications, including restricting nations to balanced budgets. Thus, there is a pressing need to assess these potential trade-offs further in more realistic modelling frameworks.

## Conclusion

In this paper, we distinctively examined the evidence of spillovers, contagion, and decoupling for 12 Asian markets, Australia, and the USA using equity market indices. We found strong evidence of changes in spillovers between these markets, with increasing evidence of growing effects over the four periods (i.e. the pre-GFC, GFC, EDC and the recent periods). The continued effects of the US markets on Asia were also apparent. There is a high degree of spillovers from China and the US, both to each other and to other Asia markets. We also found strong evidence of positive and negative spillovers during the different periods reflecting shocks transmission and absorption.

Contagion was estimated through the portfolio mimicking factor framework using moment conditions. The findings showed strong evidence of both contagion and decoupling effects using the US as the global mimicking factor. The Asian markets showed evidence of decoupling from the shocks in the US market during the GFC period. In particular, the Asian markets were less affected by turmoil in the US market than would have been anticipated by the degree of spillovers evident in the pre-GFC period. The European debt crisis and the most recent periods also showed signs of change in the transmission of events via the contagion mechanism, although these effects did not bring the transmissions back to pre-GFC levels.

A key aspect of this study was to detect vulnerability in the financial market focusing on distinguishing between contagion and spillovers. The findings provide strong evidence of the changing vulnerability among the Asian and global markets. The results have direct relevance to the policy makers and regulators by giving guidance in designing appropriate policies to monitor financial markets thus promoting financial stability.

## Footnotes

1 Asia’s growing importance is discussed in Chowdhury et al. (2019).

2 Dungey et al. (2018b) provides further technical details on GHD, and see Dungey and Renault (2018) for more information on contagion methodology.

3 The choice of *p* in the empirical section is based on Akaike information criteria (AIC). We used $p = 2$.

4 Billio et al. (2012) took the alternative route of pre-filtering their data for GARCH properties before examining links between them. We did not follow this approach because we wanted to exploit how the relationships between the series move through periods of changing volatility.

5 With the consequence the first part of the data do not provide empirically analytical decompositions (Hualde and Robinson 2010).

6 See Dungey and Fry-Mckibbin (2019) and Fackler and McMillin (1998) for more details.

7 For example, trend given by $\beta _{i} = \gamma _{i,o} + \gamma _{i,1} t$.

8 See Dungey and Renault (2018) for more details.

9 The financial crisis in Cyprus was also resolved in 2014 and was relatively minor compared with the conditions experienced earlier in the European debt crisis period.

10 See, for example, the analysis of CDS data in Dungey et al. (2019c).

11 See for example the literature on diabolical loops.

12 Contagion and decoupling refer to the distinct and abrupt positive and negative changes in the transmission of shocks between markets after controlling for what would be expected from normal spillover effects. That is, they are transmissions that would not have been expected ex-ante based on existing historical relationships.

13 See Botman et al. (2013) for evidence on Japanese markets acting as a safe haven.

14 See Dungey and Renault (2018) for further details on a multivariate implementation.

## References

[CR1] Acemoglu D, Ozdaglar A, Tahbaz-Salehi A (2015). Systemic risk and stability in financial networks. Am Econ Rev.

[CR2] ADB (2017) The era of financial interconnectedness: How can Asia strengthen financial resilience? Asian Economic Integration Report

[CR3] Agénor PR, Kharroubi E, Gambacorta L, Lombardo G, Pereira da Silva LA (2017) The international dimensions of macroprudential policies. BIS working papers no. 643

[CR4] Aizenman J, Jinjarak Y, Park D (2015) Financial development and output growth in developing Asia and Latin America: a comparative sectoral analysis. NBER working paper no. 20917

[CR5] Allen WA, Wood G (2006). Defining and achieving financial stability. J Financ Stab.

[CR6] Arslanalp MS, Liao W, Piao S, Seneviratne M (2016) China’s growing influence on Asian financial markets. IMF working paper WP/16/173

[CR7] Avdjiev S, Bruno V, Koch C, Shin HS (2019). The dollar exchange rate as a global risk factor: evidence from investment. IMF Econ Rev.

[CR8] Baur DG, Fry RA (2009). Multivariate contagion and interdependence. J Asian Econ.

[CR9] Baur D, Schulze N (2005). Coexceedances in financial markets$$-$$a quantile regression analysis of contagion. Emerg Mark Rev.

[CR10] Beirne J, Caporale GM, Schulze-Ghattas M, Spagnolo N (2010). Global and regional spillovers in emerging stock markets: a multivariate GARCH-in-mean analysis. Emerg Mark Rev.

[CR11] Billio M, Getmansky M, Lo AW, Pelizzon L (2012). Econometric measures of connectedness and systemic risk in the finance and insurance sectors. J Financ Econ.

[CR12] Botman MD, de Carvalho Filho MI, Lam MW (2013) The curious case of the yen as a safe haven currency: a forensic analysis. IMF working paper WP/13/228

[CR13] Busetti F, Harvey A (2010). When is a copula constant? A test for changing relationships. J Financ Econom.

[CR14] Caporin M, Pelizzon L, Ravazzolo F, Rigobon R (2018). Measuring sovereign contagion in Europe. J Financ Stab.

[CR15] Chiang TC, Jeon BN, Li H (2007). Dynamic correlation analysis of financial contagion: evidence from Asian markets. J Int Money Financ.

[CR16] Chowdhury B, Dungey M, Kangogo M, Sayeed MA, Volkov V (2019) The changing network of financial market linkages: The Asian experience. Int Rev Financ Anal

[CR17] Demirer M, Diebold FX, Liu L, Yilmaz K (2018). Estimating global bank network connectedness. J Appl Econom.

[CR18] Diebold FX, Yilmaz K (2009). Measuring financial asset return and volatility spillovers, with application to global equity markets. Econ J.

[CR19] Diebold FX, Yilmaz K (2012). Better to give than to receive: predictive directional measurement of volatility spillovers. Int J Forecast.

[CR20] Diebold FX, Yilmaz K (2014). On the network topology of variance decompositions: measuring the connectedness of financial firms. J Econom.

[CR21] Diebold FX, Yilmaz K (2015). Trans- Atlantic equity volatility connectedness: US and European financial institutions, 2004–2014. J Financ Econom.

[CR22] Dungey MH, Harvey J, Siklos PL, Volkov V (2018) Signed spillover effects building on historical decompositions. Tasmanian school of business and economics discussion paper series no. 2017–2011

[CR23] Dungey MH, Kangogo M, Volkov V. (2019) Changing vulnerability in Asia: contagion and systemic risk. ADB economics working paper series no. 583 10.1007/s00181-022-02322-5 PMC9638451 36373092

[CR24] Dungey M, Renault E (2018). Identifying contagion. J Appl Econom.

[CR25] Dungey M, Fry R, Martin VL (2004). Currency market contagion in the Asia-Pacific region. Aust Econ Pap.

[CR26] Dungey M, Fry R, González-Hermosillo B, Martin VL (2005). Empirical modelling of contagion: a review of methodologies. Quant Financ.

[CR27] Dungey M, Milunovich G, Thorp S, Yang M (2015). Endogenous crisis dating and contagion using smooth transition structural GARCH. J Bank Financ.

[CR28] Dungey M, Khan F, Raghavan M (2018). International trade and the transmission of shocks: the case of ASEAN-4 and NIE-4 economies. Econ Model.

[CR29] Dungey M, Harvey J, Volkov V (2019). The changing international network of sovereign debt and financial institutions. J Int Financ Mark Inst Money.

[CR30] Dungey M, Fry-Mckibbin R, Volkov V (2019) Transmission of a resource boom: the case of Australia. Oxford Bull Econ Stat

[CR31] Dungey M, Zhumabekova D (2001) Testing for contagion using correlations: some words of caution. Federal Reserve Bank of San Francisco

[CR32] Elekdag S, Rungcharoenkitkul MP, Wu MY (2012) The evolution of Asian financial linkages: key determinants and the role of policy. IMF working paper wP/12/262

[CR33] Fackler JS, McMillin WD (1998). Historical decomposition of aggregate demand and supply shocks in a small macro model. South Econ J.

[CR34] Forbes KJ, Rigobon R (2002). No contagion, only interdependence: Measuring stock market comovements. J Financ.

[CR35] Giannetti M, Laeven L (2015). Local ownership, crises and asset prices: evidence from US mutual funds. Rev Financ.

[CR36] Haldane A (2009) Rethinking the financial network. Speech delivered at the Financial Students Association, Amsterdam

[CR37] Hualde J, Robinson PM (2010). Semiparametric inference in multivariate fractionally cointegrated systems. J Econom.

[CR38] Hwang E, Min H-G, Kim B-H, Kim H (2013). Determinants of stock market comovements among US and emerging economies during the US financial crisis. Econ Model.

[CR39] Kaminsky GL, Reinhart CM (2003) The center and the periphery: the globalisation of financial turmoil. NBER working paper no. 9479

[CR40] Kaminsky GL, Reinhart CM (2002). Financial markets in times of stress. J Dev Econ.

[CR41] Kim B-H, Kim H, Lee B-S (2015). Spillover effects of the US financial crisis on financial markets in emerging Asian countries. Int Rev Econ Financ.

[CR42] Lucking B, Bloom N, Van Reenen J (2018) Have R &D spillovers changed? NBER working paper no. 24622

[CR43] Mobarek A, Muradoglu G, Mollah S, Hou AJ (2016). Determinants of time varying co-movements among international stock markets during crisis and non-crisis periods. J Financ Stab.

[CR44] Rigobon R (2002). The curse of non-investment grade countries. J Dev Econ.

[CR45] Sander H, Kleimeier S (2003). Contagion and causality: an empirical investigation of four Asian crisis episodes. J Int Financ Mark Inst Money.

[CR46] Sewraj D, Gebka B, Anderson RDJ (2018). Identifying contagion: a unifying approach. J Int Financ Mark Inst Money.

[CR47] Van Rijckeghem C, Weder B (2001). Sources of contagion: Is it finance or trade?. J Int Econ.

[CR48] Yilmaz K (2010). Return and volatility spillovers among the East Asian equity markets. J Asian Econ.

